# Testicular Arteriovenous Malformation: Gray-Scale and Color Doppler Ultrasonography Features

**DOI:** 10.1155/2011/876206

**Published:** 2011-07-13

**Authors:** Fatih Gulsen, Ismail Mihmanli, Fatih Kantarci, Abdulkadir Eren, Suleyman Onder Ataus

**Affiliations:** ^1^Department of Radiology, Cerrahpasa Medical Faculty, Istanbul University, Kocamustafapasa, 34098 Istanbul, Turkey; ^2^Department of Urology, Cerrahpasa Medical Faculty, Istanbul University, Kocamustafapasa, 34098 Istanbul, Turkey

## Abstract

Intratesticular arteriovenous malformations (AVMs) are extremely rare benign incidental lesions of the testis. Ultrasonography (US) generally reveals a hypoechoic solid mass within the testicular parenchyma. We describe a patient with intratesticular AVM which was found incidentally during workup for infertility. The gray-scale and Doppler US appearance of an intratesticular AVM and the differential diagnosis have been presented. Based on the gray-scale, US appearance differentiation from malignant testicular tumors is difficult. Doppler US examination aids in the diagnosis by demonstrating the vascular nature of the tumor.

## 1. Introduction

Testicular cancer is the most common cancer in men aged 20–34 years and accounts for 1% of all malignancies in men. Gray-scale ultrasound (US) is the standard imaging technique used to identify testicular carcinoma and nearly 100% sensitive for detection of testicular tumors [1]. One of the many benefits of US examination in the diagnosis of testicular cancer is the differentiation of intratesticular from extratesticular lesions. The majority of extratesticular masses are benign and intratesticular masses are more likely to be malignant [1]. There are a variety of benign intratesticular processes, such as hematoma, orchitis, abscess, infarction, and granuloma, which mimic testicular malignancy and must therefore be considered in the differential diagnosis. It is important to be familiar with their US appearance and to closely correlate US findings and patient history to avoid unnecessary interventions. Color Doppler and power Doppler US demonstrate increased vascularity in the majority of malignant tumors and help to better define testicular involvement [2]. We present the gray-scale and color Doppler US findings in a case of intratesticular arteriovenous malformation (AVM) which was incidentally diagnosed during work-up for infertility.

## 2. Case Presentation

**Figure 1:**
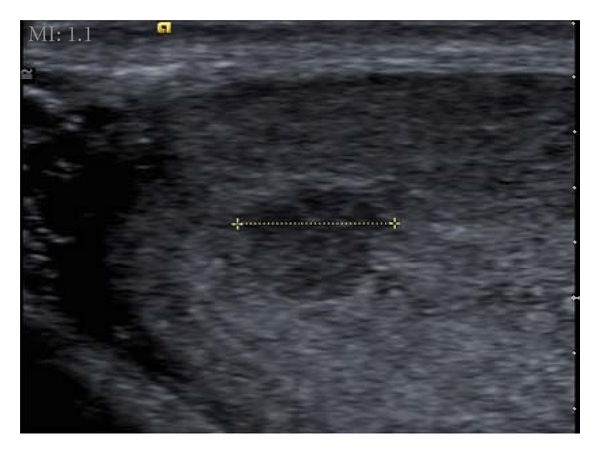
Gray-scale ultrasound image reveals a hypoechoic solid mass within the left testis.

**Figure 2:**
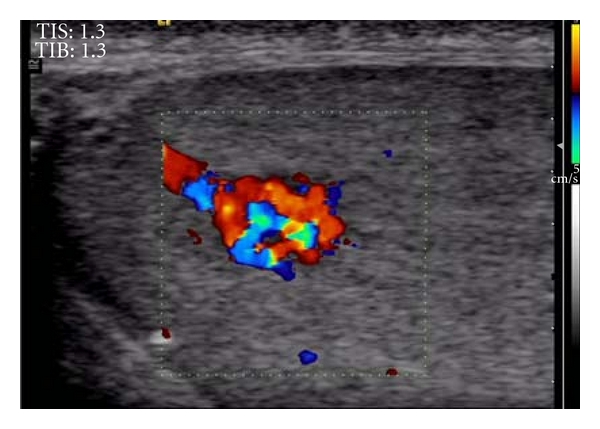
Color Doppler ultrasound demonstrates prominent vessels that entirely involve the mass.

**Figure 3:**
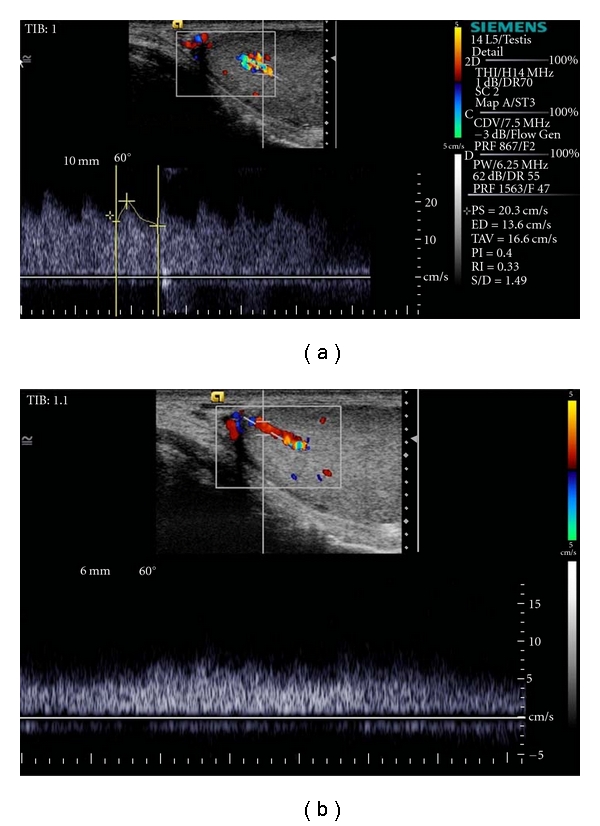
Spectral Doppler ultrasound depicts arterial flow in the feeding pedicle (a) and pulsatile venous flow in the drainage vessel (b).

A 26-year-old man was admitted to our department for sonographic evaluation of the scrotum due to infertility. On physical examination, both testes were intrascrotal, normal sized, there was no palpable lesion, and no clinical evidence of varicocele. A sperm count revealed an oligospermia (sperm count: 9400000/mL). FSH level was in the upper limit of normal. The *α*-fetoprotein and human chorionic gonadotropin levels were within normal limits. Gray-scale images showed a well circumscribed, 5.7 mm sized hypoechoic round lesion within the left testis (Figure 1). Color Doppler US demonstrated that the lesion consisted almost entirely of multiple tortuous enlarged vessels (Figure 2). There were 2 dilated vessels on both sides of the vascular mass. One of them was arterial in nature and displayed high flow velocities with a peak systolic velocity of 20.3 cm/s and end diastolic velocity of 13.6 cm/s (Figure 3(a)). The resistive index was 0.33 and pulsatility index was 0.40. The vessel on the other end showed pulsatile venous flow (Figure 3(b)) on spectral Doppler US. Based on the gray-scale and color Doppler US examinations, an arteriovenous malformation of the testis was diagnosed. US findings were not correlated to the patient's fertility status. The patient refused surgery for the mass. Because of the small size of the vascular mass, invasiveness, and risk of radiation, no angiographic imaging and endovascular interventional procedure were performed. During the followup period, there were no changes in patient's clinical and radiological findings.

## 3. Discussion

AVMs involving the lower urinary tract are uncommon as opposed to AVMs located in the Central Nervous System. Arteriovenous malformations of the spermatic cord and testis are benign lesions consisting of complex tangles of enlarged dilated arteries and veins without intervening capillaries. They can present as either painless paratesticular masses or as incidental findings during evaluation for infertility or as combination of both infertility and scrotal swelling and rarely as recurrent acute scrotal pain [3].

Most intratesticular masses are malignant and ultrasonography is the method of choice in the determination of the mass nature (cystic or solid), size, and vasculature. Besides primary testicular tumors a number of cystic appearing benign lesions such as testicular cysts, tubular ectasia of the rete testis, intratesticular varicocele, and solid-appearing benign lesions such as lipomas, hemangiomas, and AVMs can be observed [1, 4, 5]. Intratesticular AVM is a rare mass lesion of the testis and it can be confused with the other intratesticular lesions. There are sporadic case reports with hypoechoic and solid appearance on gray-scale US [4, 6, 7]. They are generally less than 1 cm in diameter. 

Of the cystic lesions, testicular cysts appear with an anechoic center, through-transmission, and without a perceptible wall [1]. There is no vascular signal on color Doppler US. Intratesticular varicocele is seen as straight and serpentine hypoechoic tubular structures within testicular parenchyma. Color Doppler US reveals venous flow within the tubular structures. The main differential of intratesticular varicocele is tubular ectasia of the rete testis in which flow is absent within the cystic structures [8]. These cystic lesions can readily be differentiated from malignant neoplasms at US. However, benign but solid-appearing lesions at US make the differentiation complicated. Intratesticular lipomas are almost always solitary, and may appear hyper- or hypoechoic avascular lesions on US [9]. Testicular hemangiomas usually are not associated with hemangiomas at other locations and categorized as capillary, cavernous, and epitheloid [10]. Hemangiomas are usually characterized by testicular enlargement with or without tenderness. Gray scale US generally shows a focal, well-defined hypoechoic mass with calcifications, and color Doppler US patterns may show variations among different types of hemangiomas, because some of them have slower flow or lesser degrees of vascular pooling. Another reported color Doppler US feature is the presence of a low resistance pattern probably representing arteriovenous shunting [10]. Intratesticular AVMs may easily be confused with hemangioma on US. Presence of extensive vascularity within the lesion with high peak systolic, end-diastolic velocities, and a low resistance flow can be seen in both AVM and hemangioma. However, demonstration of a draining vein is characteristic for an AVM as was in our case [6]. 

Use of color and power Doppler US in malignant tumors of the testis help to better define testicular involvement [1]. The presence of hypervascularity is not specific enough for a diagnosis of malignancy, and it may be difficult to demonstrate increased blood flow in small tumors. On the other hand, color Doppler US is an extremely useful method in differentiating benign lesions. Intratesticular AVM is one of the lesions that color Doppler US aids in the diagnosis, since the gray-scale US findings closely resemble a malignant tumor. 

Magnetic resonance (MR) imaging is an important imaging technique in the evaluation of scrotal masses, providing a useful adjunct to ultrasonography (US). MR imaging allows tissue characterization, with its signal intensity properties allowing detection of fat, blood products, granulomatous tissue, and fibrosis. MR imaging performed after intravenous administration of gadolinium-based contrast material allows more accurate assessment of the vascularity of testicular lesions than color Doppler US does. The pattern of enhancement of scrotal lesions can also be evaluated. MR can also help localize the lesion as intra- or extratesticular and can clearly identify an undescended testis. The main differential diagnosis of an AVM, if all other possibilities have been excluded, is that of a hemangioma. The MRI appearance of hemangioma is poorly documented. Essig et al. reported the MRI findings of a capillary hemangioma in a 26-year-old man [11]. The tumor demonstrated homogenous low signal intensity on proton density and T2-weighted images compared to normal testicular tissue. On T1-weighted images, the mass appeared almost isointense to the rest of the testis and could not be delineated.

In conclusion, intratesticular AVMs are extremely rare lesions of the testis with solid and hypoechoic appearance on gray-scale US resembling a malignant neoplasm. Color Doppler US aids in the diagnosis by showing the vascular nature of the lesion and the prominent venous drainage.
